# ER proteostasis disturbances in Parkinson's disease: novel insights

**DOI:** 10.3389/fnagi.2015.00039

**Published:** 2015-03-27

**Authors:** Gabriela Mercado, Valentina Castillo, Rene Vidal, Claudio Hetz

**Affiliations:** ^1^Faculty of Medicine, Biomedical Neuroscience Institute, University of ChileSantiago, Chile; ^2^Program of Cellular and Molecular Biology, Institute of Biomedical Sciences, University of ChileSantiago, Chile; ^3^Neurounion Biomedical Foundation, CENPARSantiago, Chile; ^4^Department of Immunology and Infectious Diseases, Harvard School of Public HealthBoston, MA, USA

**Keywords:** UPR signaling pathways, Parkinson disease, gene therapy, preclinical models, dopaminergic neurons, ER stress

## Introduction

Parkinson's disease (PD) is characterized by the selective loss of dopaminergic neurons of the substantia nigra pars compacta (SNpc). Proteostasis impairment at the level of the endoplasmic reticulum (ER) is emerging as a driving factor of dopaminergic neuron loss in PD. ER stress engages the activation of an adaptive reaction known as the unfolded protein response (UPR) to recover proteostasis or trigger apoptosis of damaged cells. The therapeutic potential of the UPR as a target has been recently validated using pharmacological and gene therapy approaches. A complex view is emerging where ER stress may have a dual role in PD, both in maintaining cell survival during initial stages of the diseases and trigger neuronal degeneration when the stress levels are sustained. Here we overview recent advances in determining the impact of ER stress to PD.

PD is a progressive neurodegenerative disease that affects movement control, characterized by the loss of dopaminergic neurons in the SNpc. In most PD cases the presence of intracellular inclusions, termed Lewy bodies (LBs) is observed, where fibrillar aggregates of αSynuclein constitute a major component. Many cellular processes are altered in PD, including redox control, mitochondrial function, autophagy/lysosomal function, protein quality control mechanisms, and vesicle trafficking, among other processes. Accumulating evidence supports disruption in the secretory pathway as a triggering factor of proteostasis dysfunction in PD, mediating in part the selective degeneration of dopaminergic neurons (Chua and Tang, 2013; Mercado et al., 2013). Importantly, in addition to PD, ER stress is emerging as a relevant driver of most common neurodegenerative diseases (Hetz and Mollereau, 2014).

ER stress activates the UPR, a complex signaling transduction pathway that mediates cellular adaptation to restore ER function (reviewed in Ron and Walter, 2007; Hetz, 2012). In this article we discuss recent insights on the significance of ER stress as a driver of dopaminergic neuron loss in PD and the potential of targeting UPR components to augment the homeostatic capacity of the ER and reduce pro-apoptotic signals.

## ER stress signaling

**Figure 1:**
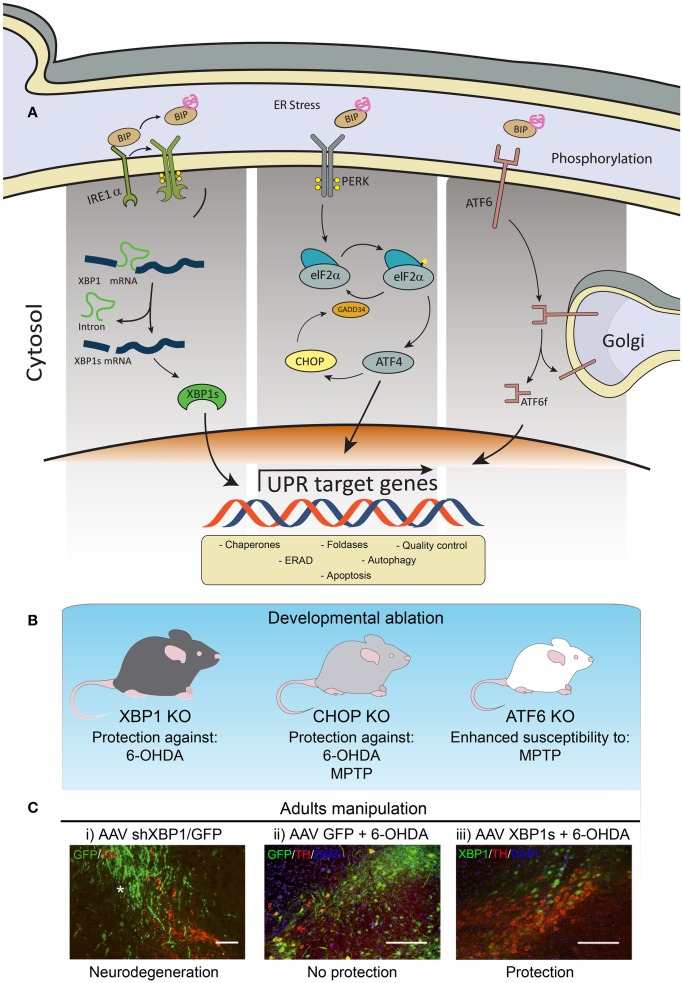
**Involvement of ER stress in PD. (A)** Schematic representation of the three branches of the UPR. **(B)** Knockout (KO) animals for XBP1, CHOP or ATF6 have been tested to manipulate the UPR in PD models. **(C)** Images modified from Valdes et al. (2014): Wild-type mice were injected into the with (i) AAV expressing an shRNA against XBP1 (shXBP1/GFP), (ii) EGFP alone, or (iii) a vector to overexpress XBP1s. One month after injection, experimental PD was induced using the 6-OHDA model to monitor dopaminergic neuron loss at the SNpc. Green: AAV transduced cells expressing GFP. Red: dopaminergic neurons stained with anti-tyrosine hydroxylase (TH). Scale bar: 200 μm.

The UPR is a signaling network mediated by the activation of three stress sensors located at the ER membrane, including inositol requiring kinase 1α (IRE1α), activating transcription factor 6 (ATF6), and protein kinase RNA-like ER kinase (PERK) (Figure 1A). These UPR transducers control the expression of a variety of genes involved in almost every aspect of the secretory pathway, resulting in a reduction in the load of misfolded proteins at the ER. Activation of the UPR improves the efficiency of protein folding and quality control mechanisms, in addition to enhance ER and Golgi biogenesis, protein secretion and the clearance of abnormally folded proteins through the autophagy and ER-associated degradation (ERAD) pathways. However, under chronic ER stress UPR sensors shifts their signaling toward induction of cell death by apoptosis (Urra et al., 2013).

IRE1α is an endoribonuclease that processes the mRNA encoding the transcription factor X-Box binding protein-1 (XBP1) which results in the expression of a more stable and active transcription factor, termed XBP1s (Ron and Walter, 2007). Upon activation, ATF6 traffics to the Golgi and undergoes subsequent proteolytic processing to release ATF6f, an active transcriptional factor (Ron and Walter, 2007). PERK is an ER-located kinase that upon activation phosphorylates the eukaryotic initiation factor 2α (eIF2α), attenuating general protein translation. In turn, eIF2α phosphorylation leads to the specific translation of activating transcription factor 4 (ATF4), which up-regulates many important genes functioning in redox control, amino acid metabolism and protein folding (Harding et al., 2003). Under chronic stress, ATF4 regulates the expression of pro-apoptotic genes such as CHOP.

## ER stress in PD

The mechanisms leading to ER stress in PD and the actual impact of the UPR on the degeneration cascade are just starting to be uncovered. A genetic screening in yeast revealed that one of the major physical targets of αSynuclein is Rab1, an essential component of the ER-to-Golgi trafficking machinery (Cooper et al., 2006; Gitler et al., 2008). Over-expression of Rab1 in animal models of PD reduced stress levels and protected dopaminergic neurons against degeneration (Coune et al., 2011). Importantly, the generation of neuronal cultures from induced pluripotent stem cells (iPSC)-derived from PD patients revealed major proteostasis alterations (Chung et al., 2013). The authors provided evidence indicating that ER stress is a salient molecular signature of human PD neurons. There are many other studies linking other PD genes with alteration of the secretory pathway, including LRRK2, Parkin, Pael-R, DJ-1, ATP13A2 (reviewed in Mercado et al., 2013), and VPS35 (Zimprich et al., 2011). These reports suggest that secretory pathway dysfunction is a common hallmark of PD, which may result in pathological levels of ER stress contributing to the etiology of the disease.

## The UPR and cell fate in PD

Genetic manipulation of essential UPR components in the context of PD had been performed only in a few studies (Figure 1B). For example, ATF6α knockout animals showed increased accumulation of ubiquitin-positive inclusions and enhanced loss of dopaminergic neurons induced by a PD-triggering neurotoxin (Egawa et al., 2011). Although ATF6 is not essential for development and survival of dopaminergic neurons in mice, this stress sensor controls the levels of the chaperone BiP and ERAD components under resting conditions in these neurons (Egawa et al., 2011). A recent study determined that ATF6 is a direct target of αSynuclein. Expression of αSynuclein was shown to inhibit the processing of ATF6 through a physical association, leading to an impaired up-regulation of ERAD genes, which sensitized cells to apoptosis (Credle et al., 2015).

We recently reported a set of *in vivo* studies uncovering the significance of the UPR transcription factor XBP1 in controlling the survival of dopaminergic neurons (Valdes et al., 2014). We found that the developmental ablation of *Xbp1* in the nervous system preconditioned dopaminergic neurons and rendered them resistant to the PD-triggering neurotoxin 6-hydroxydopamine (6-OHDA) (Figure 1B). This neuroprotective effect was accompanied by the up-regulation of several UPR effectors in the SNpc of animals in the absence of pro-apoptotic markers such as *Chop*. This phenotype correlated with the presence of poly-ubiquitinated proteins and large inclusion bodies in dopaminergic neurons of XBP1 deficient animals, resembling the classical alterations observed in PD. Remarkably, dopaminergic neurons were prompt to undergo proteostasis alterations in the absence of XBP1, a phenomenon not observed in other brain areas including cortex, striatum, or spinal cord (Hetz et al., 2009; Valenzuela et al., 2012; Vidal et al., 2012; Valdes et al., 2014). We proposed that developmental targeting of XBP1 provides neuroprotection through an “ER-hormesis” mechanism where the occurrence of mild non-lethal ER stress engages an adaptive response that sustains neuronal function in the absence of XBP1, which also renders dopaminergic neurons more resistance to a PD-inducing stimulus. In agreement with this concept, establishment of an ER-hormesis condition (Matus et al., 2012) by the administration of low doses of the ER stress agent tunicamicyn on a rodent and fly model of PD selectively engaged adaptive UPR signaling events involving the expression of XBP1s (Fouillet et al., 2012).

Since genetic manipulations during development can lead to compensatory mechanisms that mask the direct biological effects of a certain gene, we then targeted XBP1 in adult animals locally at the SNpc (Valdes et al., 2014). Knocking down XBP1 resulted in chronic ER stress involving the up-regulation of *Chop*, causing spontaneous neurodegeneration of dopaminergic neurons (Figure 1C). These results highlight the importance of XBP1 in sustaining dopaminergic neuron function and viability, reinforcing the concept that ER stress is a factor underling their differential neuronal vulnerability. Therapeutic strategy to artificially engage a UPR adaptive program has been developed to pre-adapt dopaminergic neurons to a PD-inducing event. Using a gene therapy approach, we delivered active XBP1s into the SNpc of adult mice using adeno-asociated viral (AAVs) vectors (Valdes et al., 2014). This strategy conferred a dramatic protection against 6-OHDA (Figure 1C), in addition to reduce striatal denervation. Similarly, a previous report also indicated that XBP1s gene transfer also protects dopaminergic neurons against the PD-inducing neurotoxin MPTP (Sado et al., 2009).

XBP1 has a conserved role in sustaining dopaminergic neuron survival. Recently, the over-expression of XBP1 was shown to protect against αSynuclein-induced dopaminergic neuron degeneration in *C. elegans*, whereas neuron-specific RNAi knockdown of *xbp1* exacerbates the neurodegeneration process (Ray et al., 2014). The unconventional splicing of XBP1 mRNA, in addition to require the endoribonuclease IRE1α, it involves the RNA ligase RTCB-1. This ligase also confers protection to dopaminergic neurons against αSynuclein overexpression in *C. elegans*, uncovering for the first time a functional relationship between XBP1 and its ligase in the regulation of proteostasis in neurons (Ray et al., 2014). XBP1 expression has been shown to be neuroprotective also when it is delivered into neural stem cells that are then transfer into the brain. This strategy, increased the survival of the graft and improved the motor performance in a rotenone-induced rat model of PD (Lihui et al., 2012). Finally, an AAV-based gene therapy strategy to enhance the folding capacity of the ER was also evaluated on a genetic model of PD (Gorbatyuk et al., 2012). Thus, increasing evidence indicates that the local modulation of the UPR in the nigrostriatal circuit may have important therapeutic potential in PD.

The UPR is a double-edged sword, cytoprotective when activated to a moderate extent, but degenerative when it is sustained over time. Markers of PERK/eIF2α activation have been found in PD post-mortem brain tissue, where nigral dopaminergic neurons displaying αSynuclein inclusion are also positive for phosphorylated PERK and eIF2α (Hoozemans et al., 2007). Deletion of the pro-apoptotic factor CHOP protects dopaminergic neurons against 6-OHDA and MPTP (Silva et al., 2005) (Figure 1B). Several strategies are now available to modulate PERK signaling in different disease contexts, including inhibitors of PERK activity, eIF2α phosphatases, and ATF4 expression (reviewed in Hetz et al., 2013). Salubrinal, a small compound that enhances eIF2α (Boyce et al., 2005), was shown to delay disease onset and attenuate motor deficits induced by αSynuclein over-expression (Colla et al., 2012). Unexpectedly, although salubrinal treatment attenuated disease symptoms, its administration did not protect dopaminergic neurons from degeneration (Colla et al., 2012). In the last 2 years, new exciting findings implicate the PERK/ATF4 signaling branch of the UPR as an interesting target to treat neurodegenerative diseases (Halliday et al., 2014). In this scenario, additional tools are available to systematically test the consequences of inhibiting the PERK pathway in PD models at the level of PERK, eIF2α, or ATF4, respectively.

## Perspective

Many important questions remain to be solved in this growing field. Since distinct UPR signaling branches could have specific and even opposite consequences on neuronal survival depending on the disease input (Hetz and Mollereau, 2014), a systematic approach is needed to determine what are the optimal components of the UPR pathway as possible targets to develop future therapeutic interventions. Gene therapy strategies are currently been developed in PD patients and the first results of phase I and II clinical trials are available showing excellent safety profiles (Coune et al., 2012). In this context, the possible therapeutic potential and side effects of delivering active UPR components into the SNpc in the long term remains to be determined in non-human primates since most of the available studies only used rapid-evolving PD rodent models. Another interesting aspect to explore in the future is the cell-non-autonomous control of the UPR in PD, which may propagate protective responses to other brain areas and tissues (Mardones et al., 2015). Overall all these novel insights have placed ER proteostasis in the center of the etiology of PD, which may translate in the near future into the development of prototypic strategies to alleviate dopaminergic neuron loss.

### Conflict of interest statement

The authors declare that the research was conducted in the absence of any commercial or financial relationships that could be construed as a potential conflict of interest.

## References

[B1] Boyce M. Bryant K. F. Jousse C. Long K. Harding H. P. Scheuner D.. (2005). A selective inhibitor of eIF2alpha dephosphorylation protects cells from ER stress. Science 307, 935–939. 10.1126/science.1101902 15705855

[B2] Chua C. E. Tang B. L. (2013). Linking membrane dynamics and trafficking to autophagy and the unfolded protein response. J. Cell. Physiol. 228, 1638–1640. 10.1002/jcp.24341 23460446

[B3] Chung C. Y. Khurana V. Auluck P. K. Tardiff D. F. Mazzulli J. R. Soldner F.. (2013). Identification and rescue of alpha-synuclein toxicity in Parkinson patient-derived neurons. Science 342, 983–987. 10.1126/science.1245296 24158904 PMC4022187

[B4] Colla E. Coune P. Liu Y. Pletnikova O. Troncoso J. C. Iwatsubo T.. (2012). Endoplasmic reticulum stress is important for the manifestations of alpha-synucleinopathy *in vivo*. J. Neurosci. 32, 3306–3320. 10.1523/JNEUROSCI.5367-11.2012 22399753 PMC3461828

[B5] Cooper A. A. Gitler A. D. Cashikar A. Haynes C. M. Hill K. J. Bhullar B.. (2006). Alpha-synuclein blocks ER-Golgi traffic and Rab1 rescues neuron loss in Parkinson's models. Science 313, 324–328. 10.1126/science.1129462 16794039 PMC1983366

[B6] Coune P. G. Bensadoun J. C. Aebischer P. Schneider B. L. (2011). Rab1A over-expression prevents Golgi apparatus fragmentation and partially corrects motor deficits in an alpha-synuclein based rat model of Parkinson's disease. J. Parkinsons Dis. 1, 373–387. 10.3233/JPD-2011-11058 23939344

[B7] Coune P. G. Schneider B. L. Aebischer P. (2012). Parkinson's disease: gene therapies. Cold Spring Harb. Perspect. Med. 2:a009431. 10.1101/cshperspect.a009431 22474617 PMC3312404

[B8] Credle J. J. Forcelli P. A. Delannoy M. Oaks A. W. Permaul E. Berry D. L.. (2015). Alpha-synuclein-mediated inhibition of ATF6 processing into COPII vesicles disrupts UPR signaling in Parkinson's disease. Neurobiol. Dis. 76, 112–125. 10.1016/j.nbd.2015.02.005 25725420

[B9] Egawa N. Yamamoto K. Inoue H. Hikawa R. Nishi K. Mori K.. (2011). The endoplasmic reticulum stress sensor, ATF6alpha, protects against neurotoxin-induced dopaminergic neuronal death. J. Biol. Chem. 286, 7947–7957. 10.1074/jbc.M110.156430 21131360 PMC3048681

[B10] Fouillet A. Levet C. Virgone A. Robin M. Dourlen P. Rieusset J.. (2012). ER stress inhibits neuronal death by promoting autophagy. Autophagy 8, 915–926. 10.4161/auto.19716 22660271 PMC3427257

[B11] Gitler A. D. Bevis B. J. Shorter J. Strathearn K. E. Hamamichi S. Su L. J.. (2008). The Parkinson's disease protein alpha-synuclein disrupts cellular Rab homeostasis. Proc. Natl. Acad. Sci. U.S.A. 105, 145–150. 10.1073/pnas.0710685105 18162536 PMC2224176

[B12] Gorbatyuk M. S. Shabashvili A. Chen W. Meyers C. Sullivan L. F. Salganik M.. (2012). Glucose regulated protein 78 diminishes alpha-synuclein neurotoxicity in a rat model of Parkinson disease. Mol. Ther. 20, 1327–1337. 10.1038/mt.2012.28 22434142 PMC3392977

[B13] Halliday M. Radford H. Mallucci G. R. (2014). Prions: generation and spread versus neurotoxicity. J. Biol. Chem. 289, 9862–19868. 10.1074/jbc.R114.568477 24860100 PMC4106307

[B14] Harding H. P. Zhang Y. Zeng H. Novoa I. Lu P. D. Calfon M.. (2003). An integrated stress response regulates amino acid metabolism and resistance to oxidative stress. Mol. Cell 11, 619–633. 10.1016/S1097-2765(03)00105-9 12667446

[B16] Hetz C. (2012). The unfolded protein response: controlling cell fate decisions under ER stress and beyond. Nat. Rev. Mol. Cell Biol. 13, 89–102. 10.1038/nrm3270 22251901

[B17] Hetz C. Chevet E. Harding H. P. (2013). Targeting the unfolded protein response in disease. Nat. Rev. Drug Discov. 12, 703–719. 10.1038/nrd3976 23989796

[B18] Hetz C. Mollereau B. (2014). Disturbance of endoplasmic reticulum proteostasis in neurodegenerative diseases. Nat. Rev. Neurosci. 15, 233–249. 10.1038/nrn3689 24619348

[B19] Hetz C. Thielen P. Matus S. Nassif M. Court F. Kiffin R.. (2009). XBP-1 deficiency in the nervous system protects against amyotrophic lateral sclerosis by increasing autophagy. Genes Dev. 23, 2294–2306. 10.1101/gad.1830709 19762508 PMC2758741

[B20] Hoozemans J. J. van Haastert E. S. Eikelenboom P. de Vos R. A. Rozemuller J. M. Scheper W. (2007). Activation of the unfolded protein response in Parkinson's disease. Biochem. Biophys. Res. Commun. 354, 707–711. 10.1016/j.bbrc.2007.01.043 17254549

[B21] Lihui S. Tianmin X. Fengzhang W. Qun L. Manhua C. (2012). X-box-binding protein 1-modified neural stem cells for treatment of Parkinson's disease. Neural Regen. Res. 7, 736–740. 10.3969/j.issn.1673-5374.2012.10.003 25737695 PMC4345654

[B22] Mardones P. Martinez G. Hetz C. (2015). Control of systemic proteostasis by the nervous system. Trends Cell Biol. 25, 1–10. 10.1016/j.tcb.2014.08.001 25174273

[B23] Matus S. Castillo K. Hetz C. (2012). Hormesis: protecting neurons against cellular stress in Parkinson disease. Autophagy 8, 997–1001. 10.4161/auto.20748 22858553 PMC3427272

[B24] Mercado G. Valdes P. Hetz C. (2013). An ERcentric view of Parkinson's disease. Trends Mol. Med. 19, 165–175. 10.1016/j.molmed.2012.12.005 23352769

[B25] Ray A. Zhang S. Rentas C. Caldwell K. A. Caldwell G. A. (2014). RTCB-1 mediates neuroprotection via XBP-1 mRNA splicing in the unfolded protein response pathway. J. Neurosci. 34, 16076–16085. 10.1523/JNEUROSCI.1945-14.2014 25429148 PMC4244473

[B26] Ron D. Walter P. (2007). Signal integration in the endoplasmic reticulum unfolded protein response. Nat. Rev. Mol. Cell Biol. 8, 519–529. 10.1038/nrm2199 17565364

[B27] Sado M. Yamasaki Y. Iwanaga T. Onaka Y. Ibuki T. Nishihara S.. (2009). Protective effect against Parkinson's disease-related insults through the activation of XBP1. Brain Res. 1257, 16–24. 10.1016/j.brainres.2008.11.104 19135031

[B28] Silva R. M. Ries V. Oo T. F. Yarygina O. Jackson-Lewis V. Ryu E. J.. (2005). CHOP/GADD153 is a mediator of apoptotic death in substantia nigra dopamine neurons in an *in vivo* neurotoxin model of parkinsonism. J. Neurochem. 95, 974–986. 10.1111/j.1471-4159.2005.03428.x 16135078 PMC3082498

[B29] Urra H. Dufey E. Lisbona F. Rojas-Rivera D. Hetz C. (2013). When ER stress reaches a dead end. Biochim. Biophys. Acta 1833, 3507–3517. 10.1016/j.bbamcr.2013.07.024 23988738

[B30] Valdes P. Mercado G. Vidal R. L. Molina C. Parsons G. Court F. A.. (2014). Control of dopaminergic neuron survival by the unfolded protein response transcription factor XBP1. Proc. Natl. Acad. Sci. U.S.A. 111, 6804–6809. 10.1073/pnas.1321845111 24753614 PMC4020088

[B31] Valenzuela V. Collyer E. Armentano D. Parsons G. B. Court F. A. Hetz C. (2012). Activation of the unfolded protein response enhances motor recovery after spinal cord injury. Cell Death Dis. 3:e272. 10.1038/cddis.2012.8 22337234 PMC3288350

[B32] Vidal R. L. Figueroa A. Court F. A. Thielen P. Molina C. Wirth C.. (2012). Targeting the UPR transcription factor XBP1 protects against Huntington's disease through the regulation of FoxO1 and autophagy. Hum. Mol. Genet. 21, 2245–2262. 10.1093/hmg/dds040 22337954 PMC3335312

[B33] Zimprich A. Benet-Pages A. Struhal W. Graf E. Eck S. H. Offman M. N.. (2011). A mutation in VPS35, encoding a subunit of the retromer complex, causes late-onset Parkinson disease. Am. J. Hum. Genet. 89, 168–175. 10.1016/j.ajhg.2011.06.008 21763483 PMC3135812

